# *Sulfobacillus harzensis* sp. nov., an acidophilic bacterium inhabiting mine tailings from a polymetallic mine

**DOI:** 10.1099/ijsem.0.004871

**Published:** 2021-07-08

**Authors:** Ruiyong Zhang, Sabrina Hedrich, Decai Jin, Anja Breuker, Axel Schippers

**Affiliations:** ^1^​ Federal Institute for Geosciences and Natural Resources, 30655 Hannover, Germany; ^2^​ Key Laboratory of Marine Environmental Corrosion and Biofouling, Institute of Oceanology, Chinese Academy of Sciences, 266071 Qingdao, PR China; ^3^​ Institute of Biosciences, TU Bergakademie Freiberg, 09599 Freiberg, Germany; ^4^​ Research Center for Eco-Environmental Sciences, Chinese Academy of Sciences, 100085 Beijing, PR China

**Keywords:** acidophiles, biomining, iron- and sulfur-oxidation, mine tailings, *Sulfobacillus*

## Abstract

A mixotrophic and acidophilic bacterial strain BGR 140^T^ was isolated from mine tailings in the Harz Mountains near Goslar, Germany. Cells of BGR 140^T^ were Gram-stain-positive, endospore-forming, motile and rod-shaped. BGR 140^T^ grew aerobically at 25–55 °C (optimum 45 °C) and at pH 1.5–5.0 (optimum pH 3.0). The results of analysis of the 16S rRNA gene sequences indicated that BGR 140^T^ was phylogenetically related to different members of the genus *Sulfobacillus*, and the sequence identities to *Sulfobacillus acidophilus* DSM 10332^T^, *Sulfobacillus thermotolerans* DSM 17362^T^, and *Sulfobacillus benefaciens* DSM 19468^T^ were 94.8, 91.8 and 91.6 %, respectively. Its cell wall peptidoglycan is A1*γ*, composed of *meso*-diaminopimelic acid. The respiratory quinone is DMK-6. The major polar lipids were determined to be glycolipid, phospholipid and phosphatidylglycerol. The predominant fatty acid is 11-cycloheptanoyl-undecanoate. The genomic DNA G+C content is 58.2 mol%. On the basis of the results of phenotypic and genomic analyses, it is concluded that strain BGR 140^T^ represents a novel species of the genus *Sulfobacillus*, for which the name *Sulfobacillus harzensis* sp. nov. is proposed because of its origin. Its type strain is BGR 140^T^ (=DSM 109850^T^=JCM 39070^T^).

## Introduction

The genus *Sulfobacillus* was first described in 1978 [1]. Currently, it includes five classified species with validly published names: *S. benefaciens* (type strain BRGM2^T^=DSM 19468^T^=ATCC BAA–1648^T^) [2], *S. thermosulfidooxidans* (type strain AT-1^T^=VKM B-1269^T^=DSM 9293^T^) [1], *S. acidophilus* (type strain NAL^T^=ATCC 700253^T^= DSM 10332^T^) [3], *S. thermotolerans* (type strain Kr1^T^=VKM B-2339^T^=DSM 17362^T^) [4] and *S. sibiricus* (type strain N1^T^=VKM B-2280^T^=DSM 17363^T^) [5]. The previously described *S. disulfidooxidans* [6] was reclassified as *Alicyclobacillus disulfidooxidans* [7]. Species of the genus *Sulfobacillus* have been tentatively assigned to *Clostridiales* Family XVII *Incertae sedis* [8]. All five species of the genus *Sulfobacillus* are moderately thermophilic or thermotolerant acidophiles [2]. They are endospore-forming Gram-stain-positive bacteria and are often found in low-pH environments, such as waste dumps/tailings at mine sites and acidic water streams [9–11]. Cells of species of the genus *Sulfobacillus* can obtain energy by oxidizing ferrous iron, elemental sulfur and sulfide minerals in the presence of small amounts of yeast extract. Owing to the sulfur- and iron-oxidizing activity, microorganisms of this genus are important in the oxidative dissolution of sulfide minerals [12–15]. For instance, the weak iron oxidation ability of *S. thermosulfidooxidans* improves chalcopyrite bioleaching by maintaining a favourable redox potential [16]; and chalcopyrite leaching by *S. thermosulfidooxidans* was not inhibited in the presence of 200 mM NaCl [17]. Here we report the characterization of strain BGR 140^T^ as the type strain of a novel species of the genus *Sulfobacillus*.

## Isolation and ecology

Mine tailings samples were obtained by coring from 15 to 26 m depths of the Rammelsberg sulfidic mine tailings in the Harz Mountains near Goslar, Germany (51°52′ N 10°25′ E) [18]. The main mineral phases of the samples were barite, quartz, pyrite, and sphalerite. The mean annual temperature at the sampling site is around 8 °C. The pH of a mixed tailings sample was 6.8 (tested by mixing 10 g of the sample with 25 ml pure water).

Bioleaching experiments at low pH in basal salts (BS) medium were carried out for metal extraction from the tailings samples [18]. A mixed enrichment culture including the natural microbial community in these samples was obtained. The BS medium was prepared according to methods detailed in a previous report [19]. The enrichment culture was obtained by incubating 100 ml BS medium with 10 % of mine tailings (containing approximately 7.5 % sulfide) in 250 ml Erlenmeyer flasks at 30 °C for a week with 180 rpm. shaking. A pure culture of strain BGR 140^T^ (NCBI Taxonomy ID: 2729629) was isolated from the enrichment culture by means of the overlay technique [20]. Briefly, an overlay of Feo solid medium was prepared according to methods described previously [21, 22]. Cells formed fried-egg-like and round orange-centred colonies on Feo solid plates. The isolate was grown in liquid medium containing 20 mM ferrous iron and 0.02 % yeast extract or 5 mM glucose and 0.02 % yeast extract at variable pH and temperature as described below.

## 16S rRNA Phylogeny

**Fig. 1.**
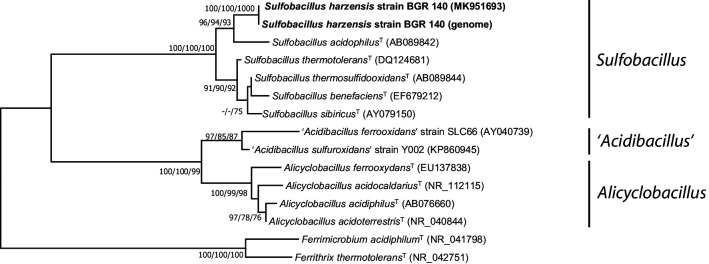
Consensus phylogenetic tree derived from partial 16S rRNA gene sequence data showing the phylogenetic relationship of *Sulfobacillus harzensis* strain BGR 140^T^ to other acidophilic members of the *Firmicutes*. Bootstrap values (>70) are shown at the respective nodes for neighbor-joining, maximum-parsimony and maximum-likelihood (left to right) trees calculated with the same sequence set. The tree was rooted with *Ferrimicrobium acidiphilum* ^T^ and *Ferrithrix thermotolerans* ^T^.

For genomic DNA extraction, approximately 5 ml microbial cultures were centrifuged for 15 min at 13 000 ***g***, and the pellet was washed twice with 10 mM Tris buffer, pH 8. DNA extraction was achieved by using the FastDNA Spin Kit for Soil (MP Biomedicals) according to a modified protocol [23]. The 16S rRNA gene of BGR 140^T^ was sequenced by Microsynth (Balgach, Switzerland). Sequences were manually edited and curated with BioEdit 7.2.5 [24]. The curated sequence was deposited in GenBank (NCBI) under the accession number MK951693. The complete 16S rRNA gene sequence was extracted from the draft genome sequence with rnammer [25] and the resulting sequence (1520 bp) showed 99.8 % identity (three mismatches out of 1493 base pairs) with the partial 16S rRNA gene sequence obtained previously. Both 16S rRNA gene sequences were compared with other 16S rRNA gene sequences available in GenBank (March 2020) using BlastN. The results of the analysis indicated that strain BGR 140^T^ was phylogenetically related to members of the genus *Sulfobacillus* with sequence identities to *S. acidophilus* ^T^, *S. thermotolerans* ^T^ and *S. benefaciens* ^T^ of 94.8, 91.8, and 91.6 %, respectively (Table S1, available in the online version of this article). The low sequence identities to species with validly published names provide proof of the status of the novel strain as representing a novel species within the genus *Sulfobacillus*. The 16S rRNA gene sequences of related type strains were downloaded from NCBI and aligned using the silva Incremental Aligner (sina v1.2.11) [26] and the silva_115NR database, followed by manual editing to remove gaps and positions of ambiguous nucleotides in mega X [27]. Phylogenetic trees were reconstructed in mega using the (i) neighbor-joining [28] and (ii) maximum-likelihood [29] algorithms based on the best-fit model of nucleotide substitution using a generalized time-reversible (GTR) model [30], and (iii) the maximum-parsimony [31] algorithm. In all cases, general tree topology and clusters were stable, and reliability of the tree topologies was confirmed by bootstrap analysis using 1000 replicate alignments. The 16S rRNA gene phylogenetic tree indicates that the genus *Sulfobacillus* forms three major clusters, while strain BGR 140^T^ forms a separate clade together with other isolates and clone sequences (Fig. 1). All three algorithms applied supported the described clustering, indicating again that strain BGR 140^T^ represents a novel species within the genus *Sulfobacillus*.

## Genome features

The whole genome of BGR 140^T^ was sequenced by the Service Centre of the German Collection of Microorganisms and Cell Cultures GmbH (DSMZ; Braunschweig, Germany). Genomic DNA extraction was carried out using MasterPure Gram Positive DNA Purification Kits from Epicentre Biotechnologies according to the manufacturer’s instructions. Libraries were prepared applying the Nextera XT DNA Library Preparation Kit (Illumina). Samples were sequenced on a NextSeq 550 Sequencing System from Illumina using a NextSeq 500/550 High Output Kit v2.5. The genome was assembled via SPAdes 3.14.0 (http://cab.spbu.ru/software/spades/) on short read genome data, which was recorded in DSMZ. After genome assembling, contigs were annotated via Prokka and finally analysed via the Type Strain Genome Server (TYGS, DSMZ) [32] and the digital DNA–DNA hybridization (dDDH) was evaluated [33]. The genome assembly is available via the NCBI BioProject: PRJNA627582. This Whole Genome Shotgun project has been deposited at DDBJ/ENA/GenBank under the accession JABBVZ000000000 and genome annotation has been done by the NCBI prokaryotic genome annotation pipeline (PGAP). The version described in this paper is version JABBVZ010000000. Details of the sequencing, assembly and genome statistics are summarized in Table 1. The genome was assembled in 397 contigs and 4647 CDS, 4721 genes and 63 tRNA were detected. The genome size was determined as 4.40 Mb. There are three genomes of type strains of members of the genus *Sulfobacillus* annotated by NCBI that are available in NCBI, *S. thermosulfidooxidans* ^T^, *S. thermotolerans* ^T^ and *S. acidophilus* ^T^. The former one has a size of 3.86 Mb and the latter two of 3.31 and 3.56 Mb, respectively (Table 1). In general, BGR 140^T^ has similar amounts of total RNA genes, rRNAs, tRNAs and ncRNAs. The genome sequence data of BGR 140^T^ were uploaded to the TYGS for a whole genome-based taxonomic analysis using the Genome-blast Distance Phylogeny (GBDP) under the algorithm ‘coverage’ and distance formula *d_5_* [33]. The phylogenetic tree was inferred with FastME 2.1.6.1 [34] from GBDP distances calculated from genome sequences and was rooted at the midpoint [35]. The overall similarities (formula *d_4_*) [33] of the genome of BGR 140^T^ with those of *S. thermosulfidooxidans* ^T^, *S. thermotolerans* ^T^ and *S. acidophilus* ^T^ are 25.5, 22.2 and 19.5 %, respectively (Table 2). The results indicate that BGR 140^T^ represents a novel species when the recommendations of a threshold value of 70 % DNA–DNA similarity for the definition of bacterial species by the ad hoc committee [36] are considered.

**Table 1.** Sequencing, assembly and annotation statistics for BGR 140^T^ and related type strains

	BGR 140^T^	* S. thermosulfidooxidans* ^T^	*S. thermotolerans^T^*	* S. acidophilus* ^T^
Sequencing technology	Illumina NextSeq	454 GS FLX, Illumina GAIIx	454; Sanger	454/Illumina
Assembly method	SPAdes 3.14.0	–	Newbler v. 2.8	Newbler v. 2.3
Annotation pipeline	NCBI PGAP	NCBI PGAP	NCBI PGAP	NCBI PGAP
Genome coverage	500×	128×	23×	30×
Contig N50 (bp)	37 136	593 49	3 317 203	–
Total length	4 395 015	3 861 015	3 317 203	3 557 831
Number of contigs	397	10	1	–
Number of proteins:	4530	3648	3121	3626
DNA G+C content (mol%)	58.20	49.70	52.4%	56.79
Genes (total)	4721	3827	3239	3695
CDS (total)	4647	3761	3172	3471
Genes (coding)	4530	3676	3121	–
Genes (RNA)	74	66	67	69
rRNAs	3, 4(16S, 23S)	6, 6 (16S, 23S)	6, 6 (16S, 23S)	5, 5 (16S, 23S)
tRNAs	63	50	51	53
ncRNAs	4	4	4	–
Pseudogenes (total)	117	85	51	155

–, Not available.

**Table 2.** Pairwise comparisons^*^ of BGR 140^T^ genome vs. type-strain genomes in Type Strain Genome Server database. Closet type strains were automatically selected by the server. All pairwise comparisons among the set of genomes were conducted using GBDP and accurate intergenomic distances inferred under the algorithm ‘trimming’ and distance formula *d_5_* [33]. 100 distance replicates were calculated each. Digital DDH values and confidence intervals were calculated using the recommended settings of the GGDC 2.1 [33]

Subject genomes	*d_0_*	C.I. *d_0_*	*d_4_*†	C.I. *d_4_*	*d_6_*	C.I. *d_6_*	Different G+C%
*Sulfobacillus acidophilus* DSM 10332^T^	13	[10.3–16.3]	19.5	[17.4–21.9]	13.3	[11.0–16.1]	1.44
*Sulfobacillus thermotolerans* Kr1^T^	12.7	[10.0–16.0]	22.2	[19.9–24.6]	13.1	[10.8–15.9]	5.79
*Sulfobacillus thermosulfidooxidans* DSM 9293^T^	12.8	[10.1–16.1]	25.5	[23.2–28.0]	13.2	[10.9–16.0]	8.54
*Bacillus nanhaiisediminis* CGMCC 1.10116^T^	12.5	[9.8–15.8]	29.1	[26.7–31.6]	12.9	[10.6–15.6]	19.75
*Anoxybacillus rupiensis* DSM 17127^T^	12.5	[9.8–15.8]	29	[26.7–31.5]	12.9	[10.6–15.6]	15.87
*Paenibacillus wulumuqiensis* Y24^T^	12.5	[9.8–15.8]	28.9	[26.5–31.4]	12.9	[10.6–15.6]	9.0
*Geobacillus thermodenitrificans* DSM 465^T^	12.5	[9.8–15.8]	28.0	[25.6–30.5]	12.9	[10.6–15.6]	9.15
*Thermaerobacter marianensis* DSM 12885^T^	12.5	[9.8–15.8]	26.2	[23.9–28.7]	12.9	[10.6–15.7]	14.29
*Caenibacillus caldisaponilyticus* B157^T^	12.5	[9.9–15.8]	23.9	[21.6–26.4]	12.9	[10.6–15.7]	6.41
*Thermaerobacter subterraneu*s DSM 13965^T^	12.5	[9.9–15.8]	22.5	[20.3–25.0]	12.9	[10.6–15.7]	13.85
*Streptomyces aidingensis* CGMCC 4.5739^T^	12.5	[9.8–15.7]	21.9	[19.7–24.4]	12.9	[10.6–15.6]	14.96
*Rhizorhabdus dicambivorans* Ndbn-20^T^	12.5	[9.8–15.8]	18.7	[16.5–21.0]	12.9	[10.6–15.6]	7.16
*Ensifer adhaerens* ATCC 33212^T^	12.5	[9.8–15.8]	18.4	[16.2–20.7]	12.9	[10.6–15.6]	4.14
*Thermomonospora amylolytica* YIM 77502^T^	12.5	[9.9–15.8]	18.4	[16.3–20.8]	12.9	[10.6–15.7]	14.61
*Blastomonas natatoria* DSM 3183^T^	12.5	[9.8–15.7]	18.3	[16.2–20.7]	12.9	[10.6–15.6]	5.18
*Paraburkholderia ginsengiterrae* DCY85^T^	12.5	[9.8–15.7]	18.2	[16.0–20.5]	12.9	[10.6–15.6]	4.3
*Paraburkholderia panaciterrae* DCY85-1^T^	12.5	[9.8–15.7]	18.2	[16.0–20.5]	12.9	[10.6–15.6]	4.17
*Sulfitobacter litoralis* DSM 17584^T^	12.5	[9.8–15.8]	17.5	[15.3–19.8]	12.9	[10.6–15.6]	0.28

*C.I. represents confidence intervals and the pairwise dDDH values between the BGT140^T^ genome and the selected type strain genomes are provided along with their C.I. for the three different GBDP formulas *d*_*0*_, *d*_*4*_ and *d*_*6*_; Formula *d_0_* (also known as GGDC formula 1) represents the length of all HSPs divided by total genome length; Formula *d_4_* (also known as GGDC formula 2) represents the sum of all identities found in HSPs divided by overall HSP length, DDH estimates based on identities/HSP length; Formula *d_6_* (also known as GGDC formula 3) represents the sum of all identities found in HSPs divided by total genome length.

†Formula *d_4_* is independent of genome length and is thus robust against the use of incomplete draft genomes.

The average nucleotide identity (ANI) was also used to evaluate the phylogenetic position of BGR 140^T^. ANI calculations were done using a web-service for ANI computation between a pair of genome sequences [37]. The ANI values of BGR 140^T^ for *S. thermosulfidooxidans* ^T^, *S. thermotolerans* ^T^, *S. acidophilus* ^T^ were 66.8, 66.1 and 68.3 %, respectively. This confirms the classification of BGR 140^T^ as representing a novel species when the species boundary cut-off of 95–96 % is considered [38, 39], although this boundary value may need further validation [40].

To predict protein encoding genes (PEGs) and ribosomal nucleic acids the RAST server [41–43] with the contigs from NCBI was used. Functional assignment was done with the RAST server with retrieved PEGs. Annotation was done by submitting the NCBI proteins and the retrieved PEGs from RAST to BlastKOALA (https://www.kegg.jp/) and eggNOG 5.0 [44]. BGR 140^T^ contains all genes putatively encoding proteins of the complete TCA cycle. Similarly, all genes putatively encoding proteins for a complete pentose phosphate cycle were detected. In addition, putative genes encoding proteins for carbon fixation, including the reductive pentose phosphate cycle (Calvin cycle), were predicted, nevertheless, the Calvin cycle seemed to be incomplete. Genes for assimilatory sulfate reduction and bacterial sulfide:quinone reductase (SQR) were detected. Genes belonging to the dsrE family (dissimilatory sulfate reduction protein E) were predicted along with TusA genes (sulfur carrier protein TusA), genes of the heterodisulfide reductase complex (hdr) and the SOR (sulfur oxygenase reductase) gene. The SOR gene of BGR 140^T^ is similar to the one from *S. acidophilus* strain TPY (83.66 % similarity, *e*-value: 0) and exhibits 49.68 % similarity to the SOR gene of *Acidianus ambivalens* DSM 3772^T^ (*e*-value: 1e^−11^). SOR seems to be present in all strains of members of the genus *Sulfobacillus* [45, 46]. A multicopper oxidase and a heme-copper terminal oxidase, which are involved in iron oxidation systems, were also predicted. In addition, 34 putative genes encoding proteins for sporulation (including the sporulation maturation protein genes and the small acid-soluble spore protein genes) were detected by analysis of the NCBI files with BlastKOALA.

## Physiology and chemotaxonomy

For characterization of growth optima, BGR 140^T^ was cultivated in BS medium containing 5 mM glucose and 0.02 % yeast extract in a 2 l bioreactor (Electrolab). Cultures were stirred at 100 rpm and aerated (0.5 l min^−1^). The bioreactor temperature was set at varying temperatures (30–55 °C) at a constant pH of 3.0, or varying pH values (2.5–4.5) at a constant temperature of 45 °C. Additional experiments were done in 100 ml shake flasks (50 ml medium at pH 1.0 and 1.5, shaken at 120 rpm) to test for pH and temperature limits for growth. Semi-logarithmic plots of cell growth by monitoring culture OD_600_ against time were used to identify exponential growth phases, and from them specific growth rates were calculated. Tests for growth with different carbon sources were done according to the methods of Johnson *et al*. [2]. In addition, the type strains of *S. thermosulfidooxidans*, *S. acidophilus*, *S. benefaciens*, *S. sibiricus* and *S. thermotolerans* were comparatively studied for their substrate utilization and anaerobic growth.

Chemotaxonomic analyses of strain BGR 140^T^ and the other described species of the genus *Sulfobacillus* were carried out by the German Collection of Microorganisms and Cell Cultures GmbH (DSMZ, Braunschweig, Germany) where cultivation of the reference strains was done on DSMZ medium at 40 °C for 2 days.

For analysis of DAP (2,6-diaminopimelic acid), cells were hydrolyzed in 4 M HCl at 100 °C for 15 h. The hydrolysates were subjected to thin-layer chromatography on cellulose plates according to a protocol described previously [47].

Cellular fatty acids were analysed after conversion into fatty acid methyl esters (FAMEs) by saponification, methylation and extraction using minor modifications of previously described methods [48, 49]. The fatty acid methyl esters mixtures were separated by gas chromatography (GC) and detected by a flame ionisation detector using Sherlock Microbial Identification System (MIS) (MIDI, Microbial ID). For identity confirmation and to resolve summed features of the MIDI analysis, the analysis was supplemented by a GC–MS) run on a GC-MS 7000D (Agilent) using a HP-5ms UI 30 m x 250 μm×0.25 μm column (Agilent) with a helium flow of 1.2 ml with an injection of 1 µl with split ratio of 7.5 : 1. The oven program was as follows: initial temperature 170 °C, ramp 3 °C min^−1^ to 200 °C, ramp 5 °C min^−1^ to 270 °C, ramp 120 °C min^−1^ to 300 °C and hold for 2 min. The inlet temperature was set to 170 °C and then linearly increased with 200 °C min^−1^ up to 350 °C and hold for 5 min. The MS parameters were set to aux temperature 230 °C, source temperature 230 °C and electron impact ionization at 70 eV with mass range of m/z 40–600 or 40–800, respectively. Peaks were identified based on retention time and mass spectra. The position of single double bounds was confirmed by a derivatization to the corresponding dimethyl disulfide adduct [50]. Branched-chain fatty acid positions, cyclo-positions and multiple double bounds were determined by derivatization to their 3-pyridylcarbinol (‘picolinyl’) and/or 4,4-dimethyloxazoline (DMOX) derivatives [51–53].

Polar lipids were extracted from freeze dried cells using a chloroform:methanol:aqueous 0.3 % NaCl mixture (1 : 2 : 0.8, by volume) and were analysed by two dimensional silica gel thin layer chromatography [54, 55]. The first direction was developed in chloroform:methanol:water (65 : 25 : 4, by volume), and the second dimension was chloroform:methanol:acetic acid:water (80 : 12 : 15 : 4, by volume). Lipid functional groups were identified using spray reagents specific for phosphate (Zinzadze), a-glycols (periodate-Schiff), and sugars (a-naphthol/H_2_SO_4_, anisaldehyde/H_2_SO_4_) [54]. Respiratory quinones were extracted from freeze dried cells using hexane and were further purified by a silica-based solid phase extraction for high-performance liquid chromatography coupled with diode-array detection and electrospray ionization tandem mass spectrometry (HPLC-DAD-MS) analysis. The mol% G+C content of genomic DNA was determined by HPLC [56].

**Fig. 2.**
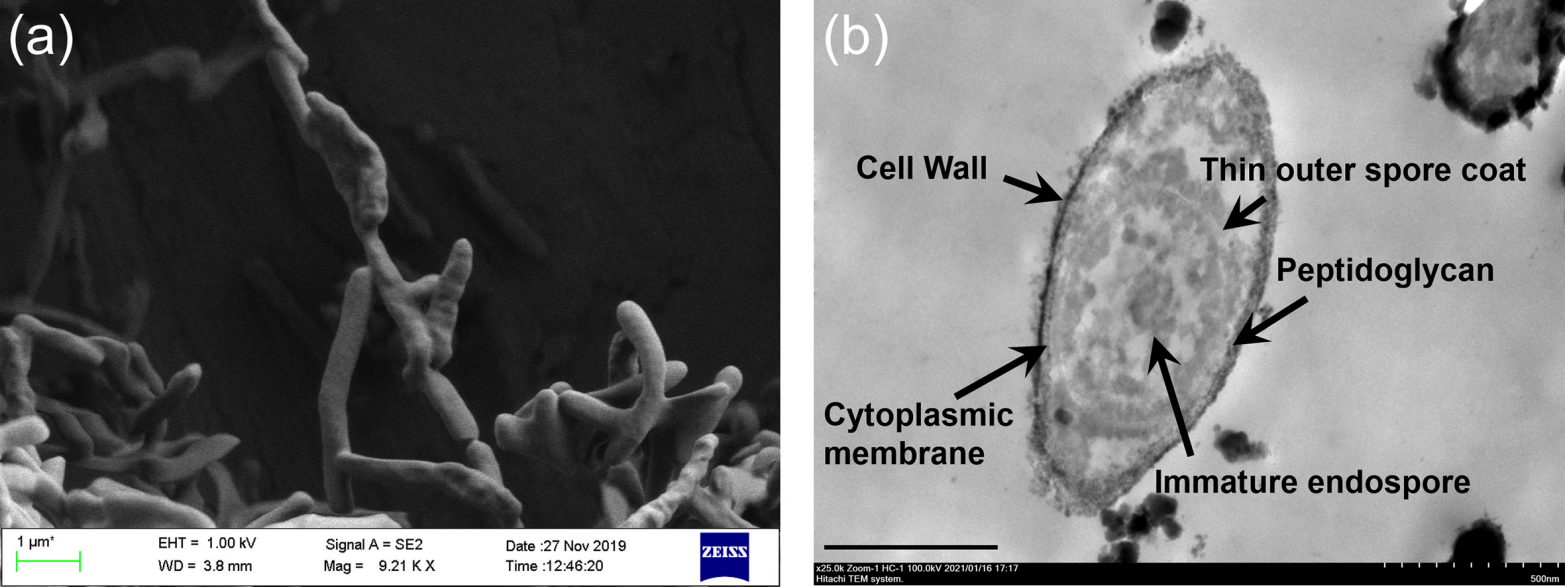
Morphology of *Sulfobacillus harzensis* strain BGR 140^T^ grown in basal salts liquid medium at pH 3.0 containing yeast extract and ferrous iron at 45 °C. (**a**) SEM observation, (**b**) TEM observation. A Zeiss Sigma 300 V P FEG scanning electron microscope operating at 1 kV was used to observe samples. For TEM, a Hitachi TEM system operated at 100 kV was used. The bar in (**b**) represents 0.5 µm.

Cell morphology was studied by using scanning electron microscopy (SEM) and transmission electron microscopy (TEM) of thin sections. Cells of BGR 140^T^ were motile, straight rods, 2.27±0.96 µm long and 0.59±0.13 µm wide as determined by SEM observations (Fig. 2a). Cells formed oval or round endospores (Figs 2b and S1). Colonies grown on Feo agar were fried-egg-like and round orange-coloured and the colony size was 0.2–1 cm after about 7 days cultivation (Fig. S2). Cells formed biofilms and aggregates on pyrite, as described for other bioleaching microorganisms [57, 58]. Cells of BGR 140^T^ grew aerobically at 25–55 °C (optimum, 45 °C) and at pH 1.5–5.0 (optimum, pH 3.0). No growth was observed at 20 °C or 60 °C.

Cell wall type of BGR 140^T^ was A1γ peptidoglycan with *meso*-diaminopimelic acid as the diagnostic diamino acid. The major polar lipids were determined to be glycolipid, phospholipids and phosphatidylglycerol (Fig. S3). The fatty acid composition of BGR 140^T^ was different to that of other species of the genus *Sulfobacillus* with validly published names. The predominant fatty acid was 11-cycloheptanoyl-undecanoate, there were also minor amounts of anteiso-C_17 : 0_, C_16 : 0_, anteiso-C_15 : 0_, C_12 : 0_, iso-C_16 : 0_ and iso-C_15 : 0_. BGR 140^T^ shared its minor fatty acids with several other type strains but 11- cycloheptanoyl-undecanoate only occurred in *S. acidophilus* and BGR 140^T^ (Table 3). The respiratory quinone of BGR 140^T^ was demethylmenaquinone (DMK) 6. DMK-6 was also the predominant respiratory quinone in cells of *S. acidophilus*, *S. benefaciens* and *S. sibiricus*. Cells of *S. thermotolerans* possessed MK-6 (80.3 %) as the main isoprenoid quinone, while a smaller amount of MK-7 (19.7 %) was also detected. In a previous study MK-7 was the only reported respiratory quinone of *S. thermotolerans* [4]. The respiratory quinones of *S. thermosulfidooxidans* included DMK-6 (39.8 %), MK-6 (45.1 %) and MK-7 (15.1 %) (Table 3). The DNA G+C content of BGR 140^T^ determined by HPLC was 58.8 mol%, close to 58.2 mol% which was estimated from the draft genome. The DNA G+C content of BGR 140^T^ was higher than that of the other strains of members of the genus *Sulfobacillus* (Table 3). Growth was tested with various substrates for BGR 140^T^ and the other described species of the genus *Sulfobacillus*. As the other five species do, BGR 140^T^ can oxidize ferrous iron, elemental sulfur and metal sulfides in the presence of yeast extract. These are typical features of species of the genus *Sulfobacillus*, which enable these acidophilic microorganisms to flourish in bioleaching environments. A suite of organic substrates including glucose, mannose, arabinose, fructose, sucrose, starch, ethanol, mannitol, glutamic acid, alanine and casein can be assimilated by cells of BGR 140^T^ (Table 3). It shares the ability to assimilate some organic substrates, e.g. glucose, mannose and sucrose, with the other five species of the genus *Sulfobacillus*. Cells are capable of anaerobic growth with ferric iron as an electron acceptor as observed for all other species of the genus *Sulfobacillus* in this study in agreement with previous data [2]. The growth pH and temperature of BGR 140^T^ fall within the range of those for species of the genus *Sulfobacillus*. These indicate that BGR 140^T^ should be classified as representing a species of the genus *Sulfobacillus*.

**Table 3.** Characteristics of strains of species of the genus *Sulfobacillus* Strains: 1, BGR 140^T^; 2, *S. benefaciens* ^T^ [2]; 3, *S. thermotolerans* ^T^ [4]; 4, *S. sibiricus* ^T^ [5]; 5, *S. acidophilus* ^T^ [3]; 6, *S. thermosulfidooxidans* ^T^ [1]. All strains were rod-shaped, endospore-forming and were able to grow on the inorganic electron donors sulfide minerals, ferrous iron and elemental sulfur and on yeast extract. Anaerobic growth with ferric iron as an electron acceptor and glucose as an electron donor was observed for all strains determined as described for *S. benefaciens* ^T^ [2]

Characteristic	1	2	3	4	5	6
Cell size	2.3±0.96×0.6±0.13	2.5±0.5×0.6±0.05	3.0±2.1×1.0±0.28	2.0±1.4×0.9±0.28	4.0±1.4×0.65±0.21	2.0±1.4×0.7±0.14
Growth pH range (optimum)	1.5–5.0 (3.0)	0.8–2.2 (1.5)	1.2–2.4 (2.0)	1.1–2.6 (2.0)	(2.0)	1.5–5.5 (1.7–2.4)
Growth temperature (optimum) (℃)	25–55 (45)	30–47 (38.5)	20–60 (40)	17–60 (55)	(45–50)	20–60 (50–55)
DNA G+C content (mol%)	58.2*	50.4–50.8	47.7–48.7	48.2	55–57	47.2–47.5
Respiratory quinones†	DMK-6 (100 %)	DMK-6 (74.4 %); MK-6 (25.6 %)	MK-6 (80.3 %); MK-7 (19.7 %)	DMK-6 (94.3 %); MK-6 (5.7 %)	DMK-6 (66.2 %); MK-6 (33.8 %)	DMK-6 (39.8 %); MK-6 (45.1 %); MK-7 (15.1 %)
Major fatty acids (>1 %) in order of their abundance†	11-cycloheptanoyl- undecanoate, anteiso-C_17 : 0_, C_16 : 0_	anteiso-C_17 : 0_, anteiso-C_15 : 0_, iso-C_16 : 0_, 8-methylpentadecanoate, iso-C_15 : 0_, iso-C_17 : 0_	anteiso-C_15 : 0_, anteiso-C_17 : 0_, iso-C_16 : 0_, iso-C_15 : 0_, iso-C_17 : 0_, C_16 : 0_	anteiso-C_17 : 0_, anteiso-C_15 : 0_, iso-C_16 : 0_, iso-C_15 : 0_, iso-C_17 : 0_, C_16 : 0_, C_15 : 0_‡	anteiso-C_15 : 0_, anteiso-C_17 : 0_, iso-C_15 : 0_, iso-C_16 : 0_, 11- cycloheptanoyl- undecanoate, iso-C_17 : 0_, C_16 : 0_, iso-C_14 : 0_, iso-C_15 : 0_ 2-OH, C_17 : 1_ω6*c*, C_15 : 0_	anteiso-C_17 : 0_, anteiso-C_15 : 0_, iso-C_16 : 0_, iso-C_15 : 0_, C_16 : 0_, C_17 : 1_ω6*c*, iso-C_17 : 0_‡
**Growth substrates** ^§^						
**Saccharides**						
Glucose	+	+	+	+‡	+	+
Fructose	+	+	+	+‡	+	+
Sucrose	+	+	+	+‡	+	+
Arabinose	+	−	+	+	+	+
Mannose	+	+	+‡	−	+‡	+
Galactose	+	+	+	−	+	+‡
Glucuronic acid	+	−	+	−	+	+
**Alcohols**						
Glycerol	+	+	−	−	+	−
Mannitol	+	+	+	−	+	−
Ethanol	+	+‡	+	+	+	+
**Organic acids**						
Acetic acid	−	−	−	−	−	+
Citric acid	−	+	+	−	+	−
Glycolic acid	−	−	−	−	−	−
Malic acid	+	−	-‡	−	+	−
Succinic acid	−	−	−	−	−	−
**Amino acids**						
Glycine	−	−	+	−	+	+
Glutamic acid	+	+	−	−	+‡	+
Histidine	−	+	+	−	+	−
Alanine	+	−	+	−	+	+
Arginine	−	−	+	−	+	+
Tryptophan	−	+	−	−	−	−
**Casein**	−	+	−	−	+	+
**Starch**	+	−	+	−	+	−

Growth was determined by increase of cell numbers via microscopic examination of cultures, and scored as: +, growth; −, no growth. Basal salt medium was supplemented with 0.008 % (w/v) yeast extract, soluble growth substrates were added in concentrations of 5 mM or 10 mM (alcohols).

*Determined by DSMZ;

†Determined by DSMZ for all strains;

‡Results not consistent with the literature.

§Data for growth substrates are our own data for all strains tested close to their particular pH and temperature optimum.

## Full-Text

## Description of Sulfobacillus harzensis sp. nov.

*Sulfobacillus harzensis* sp. nov. (harz.en′sis. N.L. masc. adj. *harzensis* of or pertaining to the Harz Mountains).

Cells are 2.27±0.96 µm long and 0.59±0.13 µm wide, motile, Gram-stain-positive rods that form subterminal or central round or oval endospores. Colonies grown on ferrous iron agar are fried-egg-like and round orange-centred and the colony size was 0.2–1 cm after about seven days cultivation. Growth occurs at 25–55 °C (optimum 45 °C) and at pH 1.5–5.0 (optimum pH 3.0). Facultative autotroph capable of autotrophic growth with elemental sulfur, ferrous iron and metal sulfides. Facultative anaerobe, capable of anaerobic growth with ferric iron as an electron acceptor. A suite of organic substrates including glucose, mannose, arabinose, fructose, sucrose, starch, ethanol, mannitol, glutamic acid, alanine and casein can be assimilated. The major polar lipids are glycolipid, phospholipid and phosphatidylglycerol. The most abundant cellular fatty acid is 11-cycloheptanoyl-undecanoate. The respiratory quinone is DMK-6. The cell wall peptidoglycan is A1γ composed of *meso*-diaminopimelic acid.

The type strain is BGR 140^T^ (=DSM 109850^T^=JCM 39070^T^), which was isolated from mine tailings in the Harz mountains, near Goslar, Germany (51°52′ N 10°25′ E). The genomic DNA G+C content of the type strain is 58.2mol%. The unassembled and assembled genome sequencing data (JABBVZ000000000) and 16S rRNA gene (MK951693) were assigned to the NCBI BioProject: PRJNA627582.

## Supplementary Data

Supplementary material 1
