# Supplementary material for: Sulfobacillus harzensis sp. nov., an acidophilic bacterium inhabiting mine tailings from a polymetallic mine
Source: Int J Syst Evol Microbiol. 2021 Jul 8;71(7):004871. doi: 10.1099/ijsem.0.004871 (PMC8489842; doi:10.1099/ijsem.0.004871)
Supplement: Supplementary material 1 [file ijsem-71-4871-s001.pdf]

Supplementary Table S1. Relatedness of 16S rRNA genes from all type strains of the genus *Sulfobacillus* and BGR 140^T (MK951693).

**Strain**	**Identity of 16S rRNA gene sequence (%)**					
	1	2	3	4	5	6
**BGR 140^T (1)**	-					
***S. benefaciens*^{T} (2)**	91.6	-				
***S. thermotolerans*^{T} (3)**	91.8	95.4	-			
***S. sibiricus*^{T} (4)**	90.6	96.8	95.6	-		
***S. acidophilus*^{T} (5)**	94.8	90.3	90.6	89.6	-	
***S. thermosulfidooxidans*^{T} (6)**	91.2	97.1	95.6	98.7	90.5	-

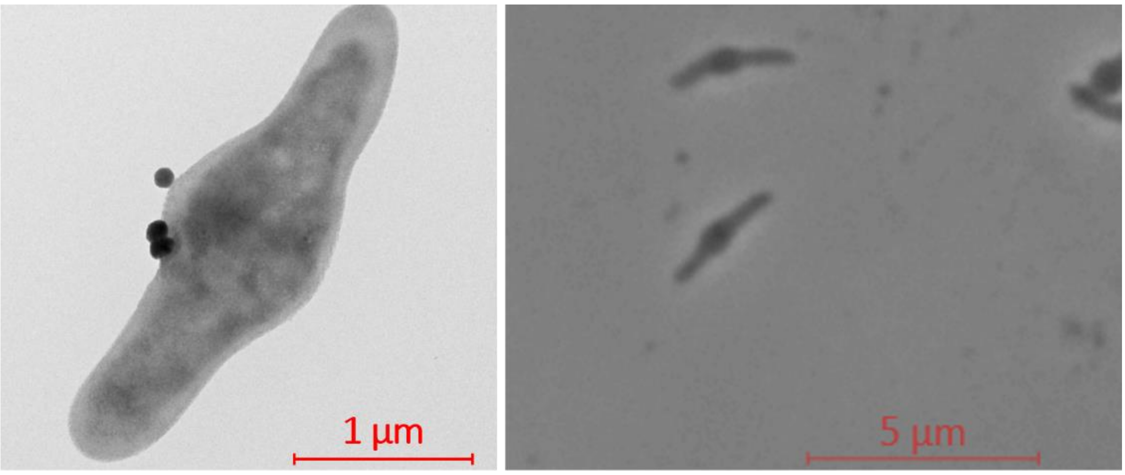

Supplementary Fig. S1. Transmission electron microscopic (left) and phase-contrast light-microscopic (right) observations of endospores in cultures of *Sulfobacillus harzensis* strain BGR 140^{T}.

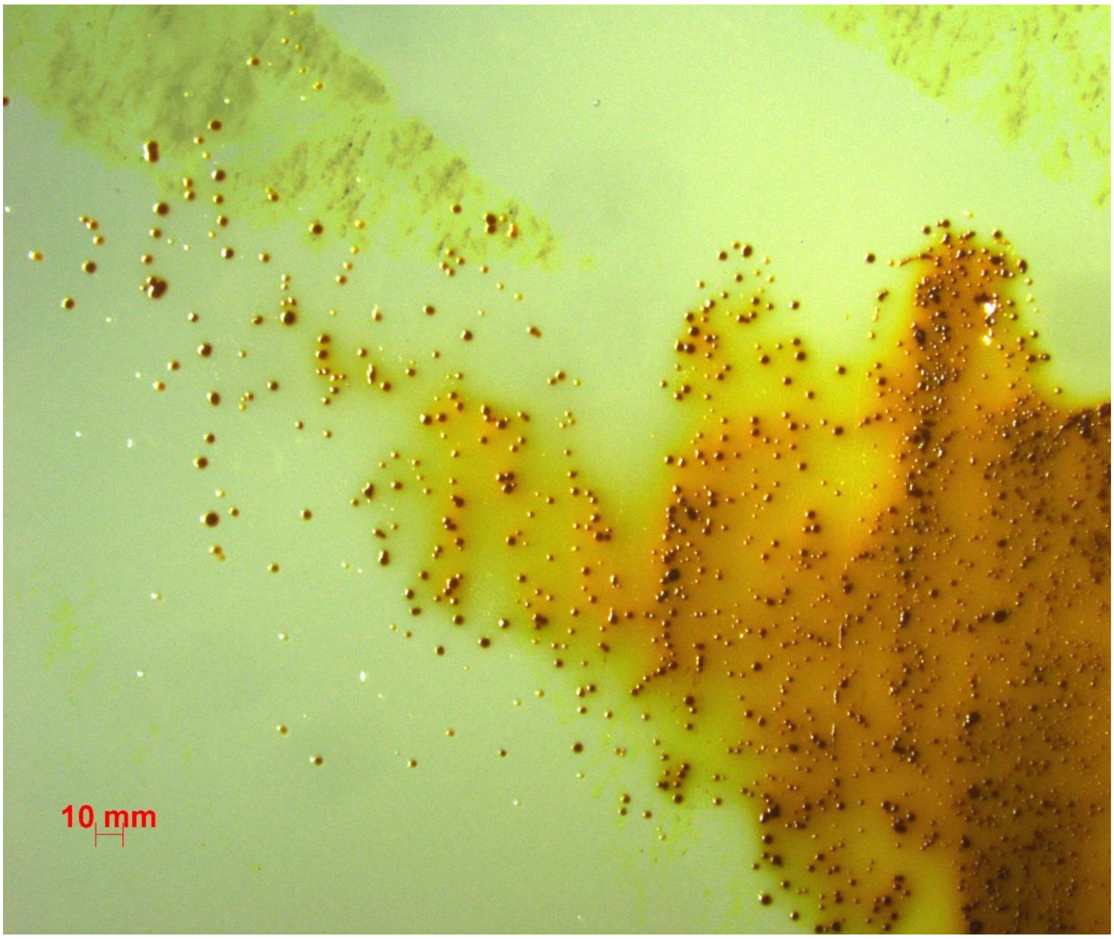

Supplementary Fig. S2. Colonies of *Sulfobacillus harzensis* strain BGR 140^T on an overlay plate of Feo solid medium.

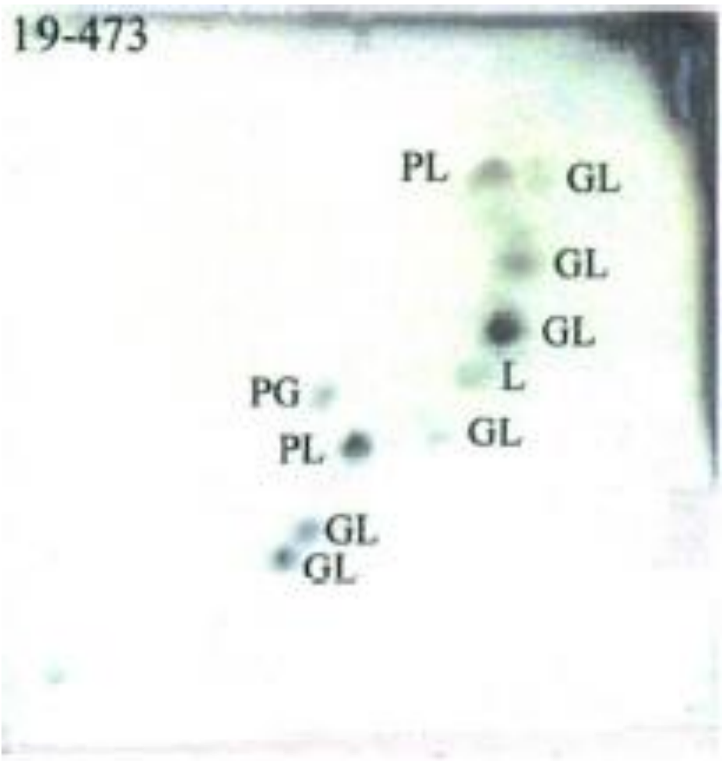

Supplementary Fig. S3. Two-dimensional thin-layer chromatography of polar lipids of the strain BGR 140^T. Polar lipids were extracted from freeze dried cells using a chloroform:methanol:aqueous 0.3% NaCl mixture (1:2:0.8, v/v) and were analyzed by two dimensional silica gel thin layer chromatography. The polar-lipid mixture was spotted into one corner (1 cm from two adjacent edges) of an aluminum-backed 10 cm x 10 cm silica gel thin-layer chromatography plate (Macherey Nagel article no. 818 135). The plates were deployed in 20 cm x 10 cm x 5 cm (internal dimensions) glass chromatography tanks lined with Whatman no. 1 filter paper. Plates were run in the first direction by using chloroform-methanol-water (65:25:4, v/v). The plates were then air dried for 30 min at room temperature and then run in the second solvent using a new chromatography chamber for the second dimension, lined with Whatman no. 1 filter paper and the solvent chloroform-methanol-glacial acetic acid-water (80:12:15:4, v/v). The various lipids were visualized by using the specific spray reagents. L Lipid; GL Glycolipid; PL Phospholipid; PG Phosphatidylglycerol.
